# Effects of second hand smoke on airway secretion and mucociliary clearance

**DOI:** 10.3389/fphys.2012.00342

**Published:** 2012-08-28

**Authors:** Yanyan Liu, Y. Peter Di

**Affiliations:** ^1^Department of Environmental and Occupational Health, University of PittsburghPittsburgh, PA, USA; ^2^Department of Physiology, Harbin Medical UniversityHarbin, China

**Keywords:** epithelium, mucus, mucociliary clearance, smoke

## Abstract

The airway acts as the first defense against inhaled pathogens and particulate matter from the environment. One major way for the airway to clear inhaled foreign objects is through mucociliary clearance (MCC), an important component of the respiratory innate immune defense against lung disease. MCC is characterized by the upward movement of mucus by ciliary motion that requires a balance between the volume and composition of the mucus, adequate periciliary liquid (PCL) volume, and normal ciliary beat frequency (CBF). Airway surface fluid (ASL) is a thin layer liquid that consists of the highly viscous mucus upper “gel” layer, and the watery lubricating lower “sol” layer. Mucus production, secretion and clearance are considered to play a critical role in maintenance of airway health because it maintains hydration in the airway and traps particulates, bacteria, and viruses. Different types of epithelial cells, including secretory cells, and ciliated cells, contribute to the MCC function. Cigarette smoke (CS) contains chemicals and particulates that significantly affect airway secretion. Active and passive CS-induced chronic obstructive pulmonary disease (COPD) is frequently associated with hyperplasia of goblet cells and submucosal glands (SMGs), thus increasing the secretory capacity of the airways that impairs MCC.

## Cigarette smoke and second hand smoke (SHS)

Cigarette smoking (CS) is the single largest cause of preventable disease, disability, and death globally. Tobacco use is a major risk factor for heart attacks, strokes, chronic obstructive pulmonary disease (COPD), and cancer. Approximately 90% of lung cancer cases in the USA are attributed to cigarette smoking. Similarly, the United States Centers for Disease Control and Prevention describes tobacco use as “the single most important preventable risk to human health in developed countries and an important cause of premature death worldwide.” CS aerosols are highly concentrated mixtures of particles and gases. This “gas/particle partitioning” is the same type of physical, gas/liquid distribution process by which an atmospheric gas, such as nitrogen, dissolves in blood. When a CS aerosol is drawn into the respiratory tract (RT), a portion of each compound of interest (e.g., nicotine) will initially be in the gas phase, and a portion will be in the particle phase. Both of these phases can serve as pathways for the delivery of the compound to RT tissues include pharyngeal, bronchial, or alveolar regions (Pankow, 2001). There are an estimated 5000 different chemicals in tobacco smoke (Borgerding and Klus, 2005; Thielen et al., 2008). Many of them are the most significant source of toxic chemicals of chemically mediated disease in humans (Ezzati and Lopez, 2003; Fowles and Dybing, 2003). Most of these compounds are not present in the tobacco plant but are formed when the cigarette burns. Many studies provide evidence of the relationship between the amount of exposure to tar or CS and the risk for head and neck, lung and skin cancers. The most known CS carcinogens include polynuclear aromatic hydrocarbons (PAH), acrolein, and nitrosamines. The gas phase of CS contains various gases including ammonia, carbon dioxide, carbon monoxide, hydrogen cyanide, and nitric oxide. The particle phase contains important constituents of CS, such as tar, nicotine, aromatic hydrocarbons, phenol, and cresol. The mean size of CS particles is 0.1–0.5 μm, so they are capable of reaching small airways (Domagala-Kulawik, 2008). In addition, radioactive carcinogens such as lead-210 (210Pb) and polonium-210 (210Po) linger in second-hand smoke (SHS), which is deeper and longer than when inhaling cigarettes especially in an indoors environment.

SHS is the inhalation of smoke, called passive smoking, or environmental tobacco smoke (ETS), by persons other than the intended “active” smoker. It occurs when CS permeates any environment, causing its inhalation by people within that environment. SHS contains largely the same components as mainstream smoke, but with varying concentrations. SHS has been shown to produce more particulate-matter (PM) pollution than an idling low-emission diesel engine and it contains not only the gas phase of exhaled smoke, but also the products of combustion of a cigarette. Therefore, persons exposed to SHS are exposed to up to 50 times higher concentration of some chemicals than smokers themselves (Domagala-Kulawik, 2008). Additionally, the smoke can also enter the body through the skin, adding an additional source of exposure.

## Health effect of SHS

Similar to active smoking, exposure to SHS is also a significant health problem concern worldwide. SHS exposure increases non-smokers' risk of cardiovascular disease and stroke by inducing several proatherosclerotic changes, including endothelial damage related to oxidative stress and inflammation, increased platelet aggregation, and increased arterial stiffness (Venn and Britton, 2007; Jefferis et al., 2010). SHS is also linked to several diseases of the lung, including cancer (Brownson et al., 2002; Hecht, 2006), and COPD (Eisner et al., 2006, 2009), asthma (Butz et al., 2011). Furthermore, SHS is associated with an increased risk of type 2 diabetes mellitus (Houston, 2006; Kowall et al., 2010) and with neurocellular changes (Slotkin et al., 2006) in infants with prenatal SHS exposure. Several studies show that causal associations exist between SHS and sudden infant death syndrome, acute respiratory infections, middle ear disease, asthma in children, and coronary heart disease as well as lung and sinus cancers in adults. Evidence is suggestive but not conclusive regarding a causal relationship between SHS and many other diseases. SHS is a major public health problem because more than 126 million people in the United States are exposed to SHS. Comprehensive bans on smoking in public lead to a reduction in overall exposure to SHS for both adults and children (Menzies, 2011), but the incidence of SHS exposure is still worrisome. SHS also contributes to the development of COPD in non-smokers and significantly impairs their airway secretion and mucociliary clearance (MCC).

## Mucociliary clearance

MCC is a self-clearing mechanism of the airway to remove inhaled pathogens and particulates. The mechanisms of bronchial secretion and MCC are critical components of lung innate immunity. Bronchial secretions are mainly produced by goblet cells and submucosal glands (SMGs), but also include small amounts of surfactant from Clara cells and other fluids that are part of the airway epithelium fluid. Cilium is present on respiratory surface epithelium, bearing hair-shaped structures on its surface (cilia). Cilia beat within a PCL layer with low viscosity for which PCL is the “sol” layer with a height that approximates the length of the outstretched cilia (about 7 μm) and keeps mucus at an optimal distance from the underlying epithelia, consequently affecting the clearance of mucus (Knowles and Boucher, 2002; Tarran, 2004). The airway epithelium fluid contains many antibacterial proteins and peptides including lysozyme, lactoferrin, and β-defensins. On the top of “sol” layer is a second viscous film of mucus “gel” that traps foreign particles and microorganisms. Acute inflammatory or toxic stimuli can promote hypersecretion of mucus mediated by a large variety of cytokines and chemokines. Within the thin fluid film of mucus, the cilia act out movements coordinated in a direction toward the pharynx. Therefore the viscous film of mucus that includes its freight is transported off in the direction of the mouth, where it is either swallowed or expelled via coughing. The “gel” layer of the airway epithelium fluid is formed mainly by water, mucins (MUC), and free proteins. Mucins are highly glycosylated macromolecules. To date, more than 21 different MUC-genes have been described, at least eight of which are expressed in the RT, although MUC5AC and MUC5B are the two main gel-forming mucins secreted in the airway. Also important for good MCC are the number, structure, activity, and coordinated movement of cilia. Function of ciliary cell is essential for MCC and ciliary cells may suspend their transport function after a short time under the adverse condition of insufficient temperature and humidity, which subsequently facilitate bacterial germinal colonization. Inhaled SHS contains PM and toxic chemicals that negatively impact MCC by increasing mucus secretion and decreasing ciliated cell numbers and ciliary beat frequency (CBF). Pulmonary infections and injury to the lung tissues in COPD patients may arise when the MCC function is compromised.

## Lung epithelial cells and MCC

**Figure 1:**
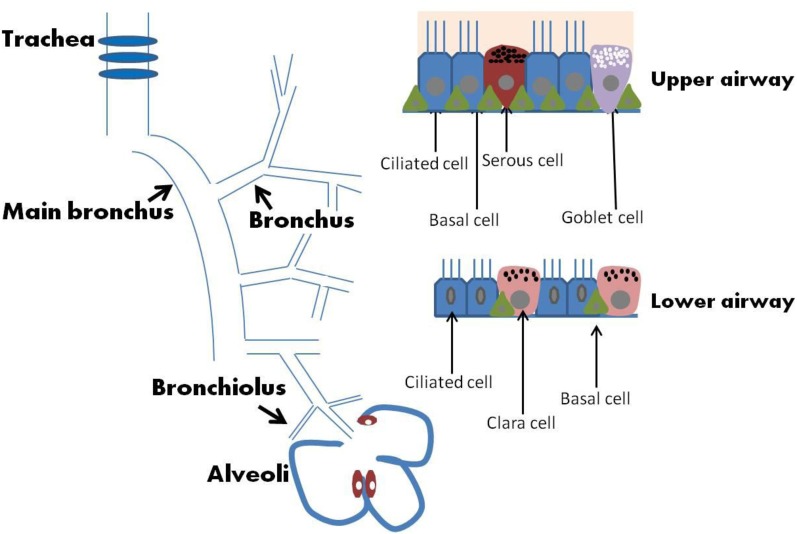
**Airway and epithelium**.

In addition to acting as a physical barrier, airway epithelium regulates fluid balance, modulates metabolism and clearance of inhaled agents, and secretes numerous mediators (Knight and Holgate, 2003). The MCC function is a coordinated action of lung epithelial cells that include a variety of cell types such as mucous, serous, goblet, ciliated, Clara, and basal cells (Figure 1). Secretory cells located in airway epithelium and SMGs are an important component of MCC mechanisms in the normal lung, and alterations in the phenotype of these cells are associated with the pathogenesis of several lung diseases. In human large airways, goblet cells of the surface epithelium, as well as serous and mucous cells of the glands, are the principal secretory cell types. Serous and mucous cells may control the properties of the mucus gel by regulating the ratio between secreted proteoglycans and mucins. Airway glandular epithelium forms an integrated structure with functionally appropriate proportion of ciliated cells, goblet cells, and basal cells. A proper ciliary movement improves valid host defense and is necessary in the cleansing processes of the airway epithelium. A brief description of major types of epithelial cells is provided in the following paragraph and summarized in Table 1. Effects of SHS on these cells and MCC will then be discussed further.

**Table 1:** **Major cell types of respiratory tract epithelium**.

**Cell type**	**Feature(s)**	**Function(s)**	**Location**	**Marker(s)**
Mucous cells	Columnar mucus-secreting cells; contain mucous electron-lucent, acidic-mucin granules	Secret mucus	Trachea, bronchi and bronchioles	MUC5AC, MUC5B
Goblet cells	Columnar mucus-secreting cells; contain mucous electron-lucent granules, which discharges apically. More abundant in human airway epithelium compared to the mouse	Contribute to airway mucus	Bronchi; small numbers in bronchioles Present in acinar region of SMGs	MUC5AC, MUC5B, SPDEF
Serous cells	Contain variable number of electron-dense granules concentrated in apical cytoplasm	Produce secretion of lower viscosity than that from mucous cells	Trachea, bronchi, present in acinar region of SMGs	Lysozyme, Lactoferrin, ZAG, SPLUNC1
Clara cells	Columnar nonciliated bronchiolar cells projecting in lumen; protuberant apical cytoplasm with large, round electron-dense secretory granules; comprise the majority of nonciliated bronchiolar cells	Secretory functions contributing to the mucous pool and maintaining extracellular lining fluid; progenitor for other bronchiolar cells	Predominantly in bronchioles	CCSP SP-A, SP-B, SP-D
Basal cells	Short cells with relatively little cytoplasm; oriented along the basement membrane; stem cells of the pseudostrafified airway epithelium	Regenerate the epithelium after loss of luminal cells and maintain it during homeostasis	Bronchi; rare in bronchioles	TP63, KRT5, PDPN and NGFR
Ciliated epithelial cells	Columnar, cuboidal, ciliated bronchial lining cells; each cell has approximately 250–300 cilia at the apical surface, and each cilium is approximately 6–7 μm long	Proximal transport of mucous stream (mucociliary escalator)	Bronchi and bronchioles	FOXJ1

***Mucous cells*** are defined by electron-lucent acidic-mucin granules that secrete mucus into the airway to trap foreign objects such as pathogens and dust particles (Jeffery, 1983). The mucous layer present in the airway from the level of the trachea to the bronchioles consists of a mixture of highly glycosylated mucin proteins. In the normal airways, there is a fine equilibrium between mucous production and clearance. However, in chronic airway inflammatory diseases, such as chronic bronchitis and asthma, mucous cell hyperplasia and metaplasia occur, which lead to excessive sputum production.

***Goblet cells*** are simple columnar epithelial cells that share multiple characteristics with mucous cells but are scattered on the surface epithelium instead of located in the secretory glands. The term goblet refers to these cells' unique “goblet-like” shape. The apical portion is shaped like a cup, as it is distended by abundant mucinogen granules; its basal portion is shaped like a stem, as it is narrow for lack of these granules. Goblet cell hyperplasia is a prominent feature in peripheral airways of smokers with both symptoms of chronic bronchitis and chronic airflow limitation (Wongsurakiat et al., 1998).

***Serous cells*** are the most abundant secretory cells in human airway glands (estimated serous/mucous cell volume ratio is 60%:40%) with variable numbers of electron-dense granules that contain large quantities of enzymes. They have irregularly shaped basally oriented nuclei, a perinuclear zone containing large quantities of rough endoplasmic reticulum (RER) required for protein (enzyme) manufacture. Serous cells are the primary defensive cells of the mucosa because they discharge bactericidal compounds that deal efficiently with pathogens (Basbaum and Jany, 1990; Joo et al., 2004). Serous cells secrete various antibacterial proteins including lysozyme, lactoferrin, secretory immunoglobin A, peroxidase, and protease inhibitors, many of which are cationic in nature.

***Clara cells*** contain electron-dense granules and are found mostly in the small airways in humans (Knight and Holgate, 2003). To regulate bronchiolar epithelial integrity and immunity, Clara cells have been shown to produce bronchiolar surfactants and specific antiproteases, such as secretory leukocyte protease inhibitor. In addition, Clara cells can produce p450 mono-oxygenases (Dewater et al., 1986), which are able to metabolize xenobiotic compounds such as aromatic hydrocarbons, which are found in cigarette smoke (CS). Clara cells produce Clara cell 10-kDa protein, Clara cell 55-kDa protein, Clara cell tryptase, galactoside-binding lectin, possibly a specific phospholipase, and surfactant proteins A, B, and D.

***Basal cells*** are highly abundant in both the upper and lower airways (Boers et al., 1998). Basal cells have been demonstrated to possess stem cell-like properties in that they can self-renew and give rise to secretory and ciliated epithelial cells in response to epithelial injuries (Hong et al., 2004). Thus, basal cells are thought to be the progenitor cells of lung epithelium and are potentially capable of differentiating and proliferating in many pathological circumstances.

***Ciliated epithelial cells*** are columnar epithelial cells with specialized ciliary modifications and are terminally differentiated cells that originate in either basal or secretory cells (Ayers and Jeffery, 1988). Usually, there are 250–300 cilia per cell and a large number of mitochondria provide energy to the cilia for MCC via proper ciliary beating. In general, cilia are sensory organelles and motile cilia in the airway epithelium are the engine for MCC. Ciliary beats provide the required physical force to move mucus and the trapped foreign particles while the CBF determines the flow speed of the movement. SHS decreases not only ciliated cells and ciliogenesis but also CBF.

## Effect of SHS on MCC

COPD is characterized by chronic obstruction of expiratory flow affecting peripheral airways, associated with chronic bronchitis (mucus hypersecretion with goblet cell and SMG hyperplasia) and emphysema (destruction of pulmonary parenchyma), together with fibrosis and tissue damage, and inflammation of the small airways (Chung, 2001; Reid and Sallenave, 2003; Hogg et al., 2004). Most COPD is caused by long-term active smoking or SHS and it has been documented that smoke significantly compromises MCC. Multiple toxins in SHS, including PM, oxidative chemicals, and organic compounds induce mucin production (Deshmukh et al., 2008) and excessive airway mucus is a hallmark feature of COPD. Airflow obstruction in COPD correlates with changes in mucin gene expression, increases in goblet-cell number and size (Innes et al., 2006), the occlusion of small airways with mucus (Sheehan et al., 1995), and expansion of the SMGs (Hogg, 2004; Hogg et al., 2004). CS-induced MCC dysfunction is complex and incompletely understood, but it involves adverse effects on the structure and function of cilia (Verra et al., 1995; Leopold et al., 2009; Tamashiro et al., 2009), activation of ErbB receptors, and proinflammatory effects that increase mucin production while decreasing mucus hydration and clearance. It is suggested that a CS-decreased MCC in COPD may lead to airway infection, which subsequently leads to inflammation and fibrosis.

One major characteristics of airway remodeling in COPD is airway goblet cells metaplasia and hyperplasia, which results in airway mucus hypersecretion. Airway goblet cell hyperplasia is a prominent pathophysiological feature of COPD and is an often-used end point in animal models of respiratory disease (Rogers, 1997). The cellular composition of the airway epithelium can be altered both by cell division and by differentiation of one cell into another (Ayers and Jeffery, 1988). In terms of goblet cell hyperplasia, differentiation is the major pathway for producing of new goblet cells, and cell division is the major carcinogenic pathway. The basal, serous and Clara cells are considered the primary progenitor cells, because they have the capacity to undergo division, followed by differentiation into “mature” ciliated or goblet cells. In specific experimental conditions such as exposure to SHS, goblet cell division contributes in part to the hyperplasia. However, differentiation of nongranulated airway epithelial cells is a major route for production of new goblet cells (Rogers, 1994; Nadel and Burgel, 2001). In experimental animals, production of goblet cells is usually at the “expense” of the progenitor cells, most notably serous and Clara cells, which decrease in number as goblet cell numbers increase. Serous-like cells and Clara cells are found in macroscopically normal bronchioles in human lung (Rogers et al., 1993). The decreased numbers of serous and Clara cell has pathophysiological importance because these cells produce a large number of anti-inflammatory, immunomodulatory, and antibacterial molecules that are vital to host defense (Basbaum et al., 1990; Singh and Katyal, 2000). Therefore, in CS-related respiratory diseases associated with airway mucus hypersecretion and impaired MCC, it seems that not only is there goblet cell hyperplasia, with associated mucus hypersecretion, but also a reduction in serous and Clara cells, with concomitant impaired potential for host defense.

It has been supported by epidemiological data that mucus hypersecretion is significantly associated with a more rapid decline in FEV1 and increased hospitalization of patients with COPD. Among all identified mucins, MUC5AC appears to be the predominant mucin in healthy human airway epithelial cells, and its expression is augmented in smokers and COPD patients (Peter Di et al., 2012). CS induces mucin expression in cultured human airway epithelial cells (Phillips et al., 2005; Cortijo et al., 2011) and MUC5AC secretion can be regulated by several inflammatory cytokines including TNF-α and IL-13. The expression level of MUC5B is approximately 20% of that seen for MUC5AC in airway epithelial cell and it is secreted mainly by mucous cells in SMGs and partially by goblet cells. Several mechanisms have been reported to be responsible for CS-induced mucus hypersecretion. First, CS could cause mucin production via epidermal growth factor receptor (EGFR) activation both *in vitro* and *in vivo* (Takeyama et al., 2001). Then, TNF-α-converting enzyme (TACE)-proligand-EGFR signal pathway could also be involved in CS-induced mucin overproduction (Shao et al., 2004). Furthermore, CS could promote goblet cell metaplasia indirectly through activation and recruitment of neutrophils and subsequent epithelial cell oxidative stress (Fischer and Voynow, 2002). *In vivo*, neutrophils and neutrophil products can cause the upregulation and release of mucin from surface epithelial cells (Takeyama et al., 1998; Voynow et al., 2004). EGF increased MUC5AC gene expression via ERK/MAP kinase but not p38/MAP kinase in NCI-H292 cells (Takeyama et al., 2000). Extracellular signal-regulated Kinase1/2 (ERK 1/2) plays an important role in airway mucus hypersecretion induced by CS in rats (Xiao et al., 2011).

In general, animal models have been essential to the development of every drug and therapeutic used to treat of COPD. One of important reasons for using animal models to tissues and cells isolated from COPD patients is that animals provide experimental settings that allow a clearer understanding of how an active immune system interacts with whole respiratory system. There are a large number of experimental researches on drugs and therapies of COPD induced by CS in laboratory animals. These include celecoxib (Roh et al., 2010), simvastatin (Lee et al., 2005), roflumilast (Martorana et al., 2005), neutrophil elastase inhibitors or neutrophil elastase knockout (Wright et al., 2002; Shapiro et al., 2003), matrix metalloprotease inhibitors (Pemberton et al., 2005), α 1-antitrypsin (Churg et al., 2003; Pemberton et al., 2006), curcumin (Suzuki et al., 2009), and overexpression of CuZnSOD (Foronjy et al., 2006), which all prevent CS-induced inflammation and emphysema. However, not all of the useful treatments in animal models have been used in humans. Actually, no one animal species can accurately model all features of human disease situations and humans normally get treated when they have more severe and sophisticated COPD than used experimentally in animal models. Current COPD animal models concentrate on emphysema rather than small airway remodeling, the key cause of airflow obstruction. It should be noted that small airway remodeling and emphysema are independent symptoms of CS that could co-exist in COPD patients.

## Conclusions

CS/SHS induces airway mucus hypersecretion and inflammation through various chemical components and signaling pathways. The significantly increased mucus production and secretion result in excess sputum that may promote airflow obstruction and inflammation. CS/SHS exposure also results in composition changes in airway secretion and impairment of cilia beating that are critical to a normal MCC. Increased mucin production, decreased luminal liquid, and impaired cilia beating motion in COPD have deleterious consequences for airway health, including mucus stasis and airway infection. CS/SHS-impaired MCC results in the increased infection rate and disease severity that could be associated with COPD exacerbations.

### Conflict of interest statement

The authors declare that the research was conducted in the absence of any commercial or financial relationships that could be construed as a potential conflict of interest.
